# When Thirst Ceases to Exist: A Case Report and Literature Review of Adipsic Diabetes Insipidus Following Coil Embolization of a Ruptured Anterior Communicating Artery Aneurysm

**DOI:** 10.7759/cureus.64207

**Published:** 2024-07-10

**Authors:** Maxim J Barnett, Goonja Patel, Patamaporn Lekprasert, Kay Win, Carlo Casipit, Osama Syed

**Affiliations:** 1 Internal Medicine, Jefferson Einstein Hospital, Philadelphia, USA; 2 Endocrinology, Jefferson Einstein Hospital, Philadelphia, USA; 3 Endocrinology, Diabetes and Metabolism, Jefferson Einstein Hospital, Philadelphia, USA; 4 Radiology, Jefferson Einstein Hospital, Philadelphia, USA

**Keywords:** vasopressin, thirst, diabetes insipidus, adipsic diabetes insipidus, adipsia

## Abstract

Diabetes insipidus is a condition characterized by inappropriately dilute urine in the setting of serum hyperosmolality. The two predominant subtypes include central (from lack of vasopressin production) and nephrogenic diabetes insipidus (from renal resistance to circulating vasopressin). A common manifestation is the significant pursuant thirst from excessive polyuria.

We present a case report and literature review of an infrequent variation of central diabetes insipidus known as adipsic (hypothalamic) diabetes insipidus, characterized by the absence of thirst, secondary to coiling of a ruptured anterior communicating artery aneurysm. Due to the loss of thirst, patients are at a heightened risk for hypernatremia and complications secondary to dehydration. Our patient's course was complicated by recurrent polyuria and hypernatremia, requiring a fixed-dose desmopressin regimen. On follow-up, only partial thirst sensation was restored. We provide a literature review to compare our case report to the scant literature available to broaden the awareness of this infrequent, perilous, manifestation.

## Introduction

Thirst is defined as the strong desire for water and is integral to human survival, with the strongest stimuli for thirst being hyperosmolality and hypernatremia [1]. Moreover, the desire for hydration can be an adverse effect of medications and may also be a clinical sign or symptom pertaining to an underlying pathological process.

Diabetes insipidus is one such pathology, characterized by excessive, dilute urine in the setting of serum hyperosmolality and has two predominant subtypes, either central (inadequate vasopressin production) or nephrogenic (renal resistance to vasopressin). Infrequently, during pregnancy, a third subtype can arise, whereby the placenta produces vasopressinase, which degrades vasopressin. Management of these conditions involves either replacement of the deficient hormone (administered as desmopressin, DDAVP), or enhancement of renal sensitivity to vasopressin.

We present a fourth subtype known as adipsic (hypothalamic) diabetes insipidus, which is akin to central diabetes insipidus with the caveat of impairment (or absence) of thirst sensation. Most commonly (as with our patient) this can occur following the management of a ruptured anterior communicating artery aneurysm and has been documented only a couple hundred times within the medical literature. We therefore provide a case report and review of the current literature to broaden the awareness of this phenomenon across all medical specialties.

## Case presentation

**Figure 1:**
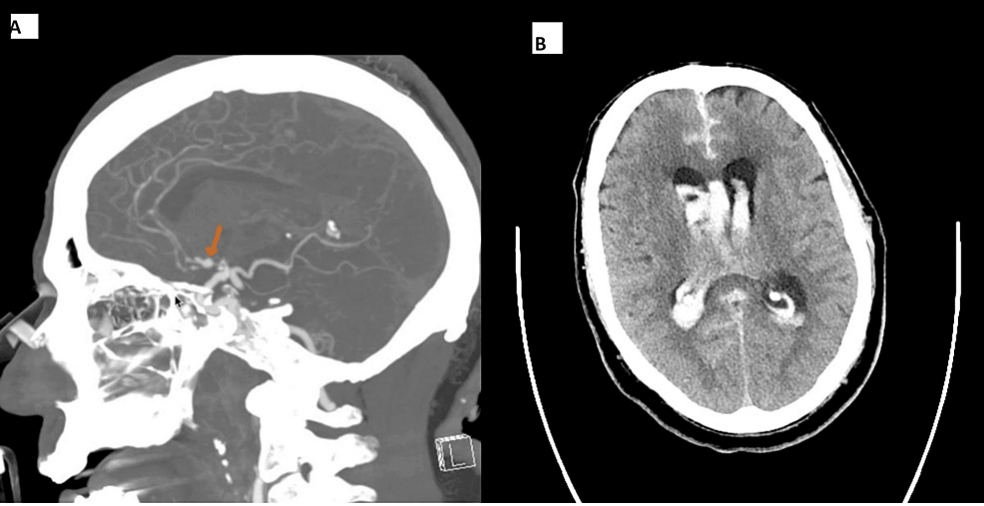
Computed Tomography Angiography of the Head and Neck (With Contrast) and Computed Tomography of the Head (Without Contrast) A: Arrow is pointing to anterior communicating artery aneurysm, demonstrating its close proximity to the hypothalamus B: Subarachnoid hemorrhage with intraventricular hemorrhages

A 65-year-old gentleman with no significant past medical history was found unresponsive by his wife at home. Emergency medical services were contacted, and he was transported to the emergency department, where he was intubated for airway protection. He underwent a computed tomography angiography scan of his head and neck, demonstrating a ruptured 7 x 4 x 4 millimeter right anterior communicating artery aneurysm (incorporating origins of A_2_ segments with antero-superior projection) leading to an acute right frontal parenchymal hematoma and subarachnoid hemorrhage (Figure 1). He was admitted to the neurosurgical unit and underwent an urgent coil embolization. During his admission, he was extubated; however, his course was complicated by dysphagia requiring the placement of a percutaneous endoscopic gastrostomy (PEG) tube, for which he received both water flushes and tube feeds. Additionally, he developed hydrocephalus requiring placement of a ventriculoperitoneal shunt (VPS), which failed, requiring subsequent placement of an external ventricular drain (EVD) and distal catheter revision of the VPS. His course was further complicated by an abdominal infection with ventriculitis requiring removal of the VPS hardware and EVD placement, with revision VPS. He improved following a course of antibiotic treatment and was transferred to acute inpatient rehabilitation three months following his initial presentation.

At the acute inpatient rehabilitation, endocrinology was consulted due to the concern for diabetes insipidus. The day prior to being seen by endocrinology, the patient was polyuric with an output of three liters and was noted to have persistent sodium levels between 144 and 147 mEq/L, alongside a urine osmolality of 475 mOsm/kg in the setting of serum osmolality of 318 mOsm/kg (additionally demonstrated orthostatic hypotension during physical therapy). These results suggested an inadequate urine concentration (suggestive of partial diabetes insipidus). A water deprivation test was not pursued given his findings suggestive of (partial) diabetes insipidus, as well as his classic history of an anterior communicating artery aneurysm clipping, and hypotension. Peculiarly, the gentleman did not complain of thirst despite being polyuric and required repetitive reminders from the nursing staff to stay hydrated; he would frequently refuse oral hydration and tube flushes stating that he was not thirsty.

The patient was started on 0.1 milligrams (mg) of oral desmopressin nightly (oral desmopressin was easier to administer by the nursing staff), and his tube flushes were decreased in frequency from 400 mL every four hours to every six hours. When re-examined by Endocrinology, his sodium had increased to 147 mEq/L, with a urine osmolality of 327 mOsm/kg and documentation of around 800 mL of urine in a few hours (polyuric); this was suggestive of inappropriately dilute urine. His oral desmopressin was increased to 0.1 mg twice daily, and his tube flushes were reverted to every four hours. 

Upon further follow-up, he remained hypernatremic, with intermittent polyuria (albeit not accurately documented) and frequent omission of tube flushes by the nursing staff. His oral desmopressin was frequently missed or taken near the timing of his tube feeds (likely impairing absorption), and he was switched to nasal desmopressin for improved absorption (one spray of 10 micrograms daily). At this time, the patient stated he had some thirst sensation return but required frequent reminders to hydrate throughout the day. His nasal demopressin was eventually increased to 20 micrograms twice daily as his PEG tube became dislodged and was no longer receiving free water flushes; in this regimen, he remained eunatremic with a stable sodium between 138 and 143 mEq/L and a decreased urine output. 

Anterior pituitary hormone panels were performed at baseline on admission, and one week later. The results demonstrated secondary hypogonadism with low testosterone, likely from traumatic brain/pituitary injury. Testosterone was not initially commenced as this was a mildly low testosterone level with a recent injury, and it was decided to wait and repeat the level in a few months in case of recovery of the gonadal axis. Two months later, however, there was no improvement, and a discussion occurred between the patient, family, and the physical medicine and rehabilitation team, and a decision was reached to avoid testosterone supplementation (Table 1).

**Table 1:** Anterior Pituitary Hormone Profile on Admission and Repeat Measurements FSH: Follicle-Stimulating Hormone; IGF-I: Insulin-Like Growth Factor I; LH: Luteinizing Hormone; mcg: Microgram; mIU: Milli-international Units, pg: Picogram; ng: Nanogram

Parameter	Reference Range	Value on Admission to Acute Inpatient Rehab	Value one Week Later	Value Two Months Later	Value Four Months Later
FSH	1.0-12.0 mIU/mL	11.8 mIU/mL	10.2 mIU/mL	-	-
LH	0.6-12.1 mIU/mL	5.7 mIU/mL	4.8 mIU/mL	-	-
Prolactin	3.5-19.4 ng/mL	5.7 ng/mL	-	-	-
Cortisol	3.7 – 19.4 mcg/dL	10.9 mcg/dL	-	-	-
Total T4	4.9-11.7 mcg/dL	7.1 mcg/dL	-	-	-
IGF-I	41.0-279.0 ng/mL	394 ng/mL	-	251 ng/mL	-
IGF-I Z Score (Male)	-2.0 - +2.0	2.8	-	1.6	
Total Testosterone	250.0-1100.0 ng/dL	-	183 ng/dL	-	173 ng/dL
Free Testosterone	35.0-155.0 pg/mL	-	27.1 pg/mL	-	-

## Discussion

Our case report adds to the scant literature on adipsic diabetes insipidus, a rare encounter with no further than a couple hundred cases posed globally (Table 2). As demonstrated, our patient had impaired thirst sensation, with only partial improvement, and required a fixed regimen of desmopressin and controlled water intake for the management of his polyuria and hypernatremia. The case presented is unique compared to other case reports or series, as the patient had a prolonged stay in acute inpatient rehabilitation (seven months), for which, it was possible to observe his disease course in a controlled environment directly.

**Table 2:** Matrix Synthesis of Literature Describing Adipsic Diabetes Insipidus ACoM: Anterior Communicating Artery; ACTH: Adrenocorticotrophic Hormone; BID: Twice per Day; DI: Diabetes Insipidus; DVT: Deep Venous Thrombosis; GH: Growth Hormone; HCTZ: Hydrochlorothiazide; IM: Intramuscular; IV: Intravenous; Melt: Sublingual, Disintegrating Tablets; mcg: Micrograms; mL: Milliliters; Pitressin: Brand Name of Desmopressin; qAM: Every Morning; qPM: Every Evening; SC: Subcutaneous; TID: Three Times per Day; TSH: Thyroid-Stimulating Hormone

Author(s)	Cause	No. of Patients	Age/Sex	Maximum Serum Sodium	Additional Findings	Medication Treatment	Outcomes
Our Case	ACoM Aneurysm	n = 1	65 Years/Male	147 mEq/L	Hypogonadotropic Hypogonadism	Intranasal Desmopressin 20 mcg BID	Partial Thirst Recovery at Four Months
Nolan and Inder 2016 [2]	ACoM Aneurysm	n = 1	36 Years/Female	156 mEq/L	-	Oral Desmopressin 200 mcg BID	-
Hassett et al. 2021 [3]	ACoM Aneurysm	n = 1	16 Years/Male	-	-	Desmopressin (Dosage Not Reported)	-
Savin et al. 2007 [4]	ACoM Aneurysm	n = 1	-	-	-	-	-
Kim et al. 2021 [5]	ACoM Aneurysm	n = 1	37 Years/Female	173 mEq/L	-	Intranasal Desmopressin 10 mcg BID	Thirst Recovered After Three Years, Able to Discontinue Desmopressin
Mavrakis and Tritos 2007 [6]	ACoM Aneurysm	n = 1	55 Years/Female	165 mEq/L	Short-Term Memory Impairment	Intranasal Desmopressin 20 mcg BID	-
Nussey et al. 1986 [7]	ACoM Aneurysm	n = 1	30 Years/Male	187 mEq/L	Thermal Regulation Impairment	-	-
Spiro and Jenkins 1971 [8]	ACoM Aneurysm	n = 1	52 Years/Female	160 mEq/L	Thermal Regulation Impairment	-	-
Nguyen et al. 2001 [9]	ACoM Aneurysm	n = 1	46 Years/Male	167 mEq/L	Short-Term Memory Impairment	Intranasal Desmopressin 20 mcg BID, HCTZ and Chlorpropamide	-
Kimura et al. 2019 [10]	ACoM Aneurysm	n = 1	43 Years/Male	168 mEq/L	Cognitive Impairment	Oral Desmopressin 0.2mg TID	-
Imai et al. 2017 [11]	ACoM Aneurysm	n = 1	38 Years/Male	165 mEq/L	-	Intranasal Desmopressin 30 mcg Daily	No Thirst Recovery, Remission of DI Allowing Suspension of Desmopressin
Tan et al. 2016 [12]	ACoM Aneurysm	n = 1	52 Years/Male	160 mEq/L	Cognitive Impairment, Seizure	Desmopressin Oral 0.2 mg BID	Thirst Recovery at Six Months
Ghosh et al. 2014 [13]	ACoM Aneurysm	n = 2	-	-	-	Desmopressin (Dose Not Reported)	-
Baylis and Robertson 1980 [14]	ACoM Aneurysm	n = 1	-	-	-	-	-
Lima et al. 2004 [15]	ACoM Aneurysm	n = 1	53 Years/Male	221 mEq/L	Short-Term Memory Impairment, Rhabdomyolysis, Renal Impairment	Oral Desmopressin 0.1 mcg Daily	-
McIver et al. 1991 [16]	ACoM Aneurysm	n = 2	(1) 39 Years/Female (2) 30 Years/Male	(1) 155 mEq/L (2) 180 mEq/L	-	(1) and (2) Intranasal Desmopressin BID (Dosages Not Reported)	-
Smith et al. 2002 [17]	ACoM Aneurysm	n = 4	(1) 39 Years/Female (2) 30 Years/Male (3) 28 Years/Male (4) 40 Years/Male	-	(1), (2) and (4): None (3) Myoclonus, Absence Seizures, Thermal Regulation Impairment	-	-
Sabzghabaei et al. 2018 [18]	ACoM Aneurysm	n = 1	57 Years/Male	167 mEq/L	-	Desmopressin (Dosage Not Reported)	Disappearance of DI and Recovery of Thirst after Two Days
Crowley et al. 2007 [19]	ACoM Aneurysm	n = 4	(1) 39 Years/Female (2) 30 Years/Male (3) 28 Years/Male (4) 40 Years/Male	(1) 155 mEq/L (2) 157 mEq/L (3) 160 mEq/L (4) 172 mEq/L	(1) and (2): None (3) Seizures and Thermal Regulation Impairment (4) Panhypopituitarism, Sleep Apnea, DVT, Obesity	Desmopressin (Route and Dosage Not Described)	-
Ball et al. 1997 [20]	ACoM Aneurysm	n = 1	28 Years/Male	191 mEq/L	-	Only Fluids	-
Cuesta et al. 2016 [21]	ACoM Aneurysm	n = 1	51 Years/Male	168 mEq/L	Partial Growth Hormone Deficiency, Hypogonadotropic Hypogonadism, Secondary Hypothyroidism	Desmopressin Oral 0.2mg TID	Recovery of Thirst after Ten Years and Vasopressin Secretion
Pearce et al. 1991 [22]	ACoM Aneurysm	n = 1	29 Years/Male	191 mEq/L	Thermal Regulatory Impairment, Short Term Memory Impairment, Seizure	Desmopressin (Dosage Not Reported)	-
Ramthun et al. 2011 [23]	ACoM Aneurysm	n = 1	56 Years/Male	157 mEq/L	Short Term Memory Impairment	-	-
Colleran et al. 2009 [24]	Craniopharyngioma	n = 1	18 Years/Male	180 mEq/L	Panhypopituitarism, Obesity	IV 1 mcg Desmopressin BID	-
Raghunathan et al. 2015 [25]	Craniopharyngioma	n = 1	11 Years/Male	164 mEq/L	Panhypopituitarism	Desmopressin 10 mcg Intranasal every 24-36 Hours	-
Pérez et al. 2019 [26]	Craniopharyngioma	n = 1	24 Years/Female	172 mEq/L	Panhypopituitarism	Desmopressin 120 mcg melt every Eight Hours	-
Pabich et al. 2019 [27]	Craniopharyngioma	n = 1	16 Years/Female	170-180 mEq/L	Panhypopituitarism	Desmopressin 1mcg SC BID	-
Skultety and Joynt 1963 [28]	Craniopharyngioma	n = 1	11 Years/Male	170 mEq/L	Hyperphagia, Thermal Regulation Impairment	Pitressin 5 Units every Other Day and 2 Units Daily	Deceased
Johnston et al. 1991 [29]	Craniopharyngioma	n = 1	26 Years/Female	167 mEq/L	Memory Impairment, Central Hypothyroidism, Secondary Adrenal Insufficiency	Subcutaneous Desmopressin (Dosage Not Described)	-
Nandi and Harrington 1978 [30]	Craniopharyngioma	n = 1	19 Years/Male	169 mEq/L	Seizures, Panhypopituitarism, Thermal Regulation Impairment, Renal Impairment	Vasopressin and Chlorpropamide	
Bode et al. 1971 [31]	Craniopharyngioma	n = 2	(1) 15 Years/Male (2) 11 Years/Male	(1) 175 mEq/L (2) 174 mEq/L	(1) Panhypopituitarism, Thermal Regulation Impairment, Obese (2) Panhypopituitarism, Obesity	(1) Vasopressin 5 units IM every Other Day (2) Vasopressin 5 units IM every Other Day	(1) Stopped Desmopressin, Started Chlorpropamide (2) Stopped Desmopressin, Started Chlorpropamide
Sinha et al. 2011 [32]	Craniopharyngioma	n = 3	(1) 15 Years/Male (2) 13 Years/Male (3) 11 Years/Male	-	(1) Panhypopituitarism (2) Panhypopituitarism (3) Panhypopituitarism, Obesity	-	(1) Thirst Recovery at 7 Months (2) Thirst Recovery at 9 Months (3) Thirst Recovery at 4 Months
Sabzghabaei et al. 2018 [18]	Craniopharyngioma	n = 1	30 Years/Female	170 mEq/L	-	Desmopressin (Dosage Not Reported)	Thirsty Recovery (Timeline Unknown)
Cuesta et al. 2016 [21]	Craniopharyngioma	n = 2	(1) 41 Years/Female (2) 29 Years/Female	(1) 172 mEq/L (2) 159 mEq/L	(1) Panhypopituitarism (2) Panhypopituitarism	(1) Desmopressin 0.2mg BID (2) Oral Desmopressin 0.2 mg TID	(1) Recovery of Thirst at 2 Years (2) Developed Unbearable Thirst 8 Years Later
Yang et al. 2024 [33]	Craniopharyngioma	n = 31	-	-	-	-	-
Miljic et al. 2014 [34]	Craniopharyngioma	n = 3	(1) 23 Years/Female (2) 20 Years/Male (3) 50 Years/Male	(1) 165 mEq/L (2) 171 mEq/L (3) 166 mEq/L	(1) Panhypopituitarism, Epilepsy, DVT (2) Panhypopituitarism, DVT, Sleep Apnea (3) Secondary Hypogonadism	-	-
Crowley et al. 2007 [19]	Craniopharyngioma	n = 4	(1) 15 Years/Female (2) 16 Years/Female (3) 36 Years/Male (4) 41 Years/Female	(1) 153 mEq/L (2) 160 mEq/L (3) 157 mEq/L (4) 162 mEq/L	(1) Panhypopituitarism (2) Panhypopituitarism, Sleep Apnea, DVT, Seizure (3) Panhypopituitarism, Seizures (4) Panhypopituitarism, Sleep Apnea, Seizures	Desmopressin (Dosage Not Reported)	-
Behan et al. 2015 [35]	Craniopharyngioma	n = 4	-	-	-	-	-
Zantut-Wittman et al. 2007 [36]	Craniopharyngioma	n = 1	34 Years/Male	173 mEq/L	Panhypopituitarism	Desmopressin Intranasal 30 mcg Daily	-
Barraso et al. 2019 [37]	Craniopharyngioma	n = 1	5 Years/Female	-	Panhypopituitarism, Seizure	-	-
Smith et al. 2002 [17]	Craniopharyngioma	n = 3	(1) 18 Years/Female (2) 16 Years/Female (3) 56 Years/Male	-	(1) Panhypopituitarism, Obesity (2) Panhypopituitarism, Obesity (3) Panhypopituitarism	-	-
Lambert et al. 2022 [38]	Craniopharyngioma	n = 2	(1) 10 Years/Male (2) 4 Years/Male	(1) 153mEq/L (2) Not reported	(1) Panhypopituitarism, DVT (2) Panhypopituitarism, DVT	Desmopressin	-
Garcia et al. 2023 [39]	Craniopharyngioma	n = 1	12 Years/Male	162.2 mEq/L	Panhypopituitarism	0.375 mg/day Desmopressin	-
Florescu et al. 2024 [40]	Craniopharyngioma	n = 1	32 Years/Male	160 mEq/L	Panhypopituitarism	Vasopressin (Dosage Not Reported)	-
Lascelles and Lewis 1972 [41]	Craniopharyngioma	n = 2	(1) 22 Years/Female (2) 20 Years/Female	(1) 170 mEq/L (2) 164 mEq/L	(1) Sleep Impairment (2) None	(1) Vasopressin Sniff (Dosage Not Reported) (2) Not Reported	-
Batista et al. 1999 [42]	Craniopharyngioma	n = 1	14 Years/Male	-	-	Desmopressin	-
Astafieva et al. 2022 [43]	Craniopharyngioma	n = 1	58 Years/Female	160 mEq/L	Panhypopituitarism	Desmopressin 0.1 mg BID (with Additional Doses with Excessive Polyuria)	-
Komatsu et al. 2001 [44]	Hypogenesis of Corpus Callosum	n = 1	16 Years/Male	181 mEq/L	Hypogonadism	Desmopressin (Dosage Not Reported)	-
Schaff-Blass et al. 1983 [45]	Dysplastic Corpus Callosum and Septum Pellucidum	n = 1	8 Months/Male	169 mEq/L	Hypothalamic Hypogonadism, Hypothyroidism	Desmopressin 0.00375 mL BID Nasal Insufflation	-
Avruskin et al. 1981 [46]	Agenesis of Corpus Callosum and Dilated Ventricles	n = 1	11 Years/Male	170 mEq/L	-	-	-
Radetti et al. 1991 [47]	Dysplastic Corpus Callosum and Dysplastic Septum Pellucidum	n = 1	4 Months/Female	167 mEq/L	Seizure	Desmopressin intranasal 0.05 mL BID	-
Takeya et al. 1996 [48]	Agenesis of Corpus Callosum	n = 1	13 Years/Female	-	-	-	-
Alhassan et al. 2021 [49]	Congenital Midline Defect	n = 1	20 Months/Female	194 mEq/L	Seizure	-	Deceased
Schalekamp et al. 1976 [50]	Holoprosencephaly	n = 1	8 Months/Female	188 mEq/L	-	Intranasal Vasopressin 2.5 mcg BID	-
Pillai et al. 2022 [51]	Septopreoptic Holoprosencephaly	n = 1	15 Years/Male	180 mEq/L	-	-	-
Booth et al. 1983 [52]	Basal Encephalocele	n = 1	31 Years/Male	158 mEq/L	Panhypopituitarism, Thermal Regulation Impairment	Desmopressin (Dosage not Reported)	-
Ohzeki et al. 1986 [53]	Agenesis of Septum Pellucidum	n = 1	16 Months/Male	172 mEq/L	-	Intranasal DDAVP 3.75 Micrograms BID	-
Masera et al. 1994 [54]	Septo-Optic Dysplasia	n = 5	(1) 9 Months/Male (2) 3 Months/Male (3) 2 Months/Female (4) 3 Months/Female (5) 5 Years/Male	-	(1) Other Endocrine Deficits, Seizure (2) TSH Deficiency, Seizures (3) ACTH Deficiency (4) Panhypopituitarism, Seizure (5) Panhypopituitarism, Seizure	Desmopressin (Dosage Not Reported)	(1) – (4) Not Described (5) Deceased
Karabay-Bayazit et al. 2002 [55]	Lobar Holoprosencephaly	n = 1	2 Years/Female	186 mEq/L	Seizure	Desmopressin (Dosage Not Reported)	-
Iraqi et al. 2018 [56]	Congenital, Unknown	n = 1	3 Years/Male	167 mEq/L	-	Desmopressin (Dosage Not Reported)	-
Janus et al. 2014 [57]	Agenesis of Corpus Callosum, Septo-Optic Dysplasia, Arachnoid Cyst	n = 1	8 Months/Male	159 mEq/L	ACTH, TSH Deficiencies, Seizure	Desmopressin (Dosage Not Reported)	-
Djermane et al. 2016 [58]	Optic Nerve Hypoplasia, Septo-Optid Dysplasia, Semilobar Holoprosencephaly	n = 7	(1) 18 Days/Male (2) 3 Days/Female (3) 5 Days/Female (4) 90 Days/Female (5) 11 Days/Male (6) 10 Days/Female (7) 5 Days/Male	(1) 158 mEq/L (2) 159 mEq/L (3) 166 mEq/L (4) 160 mEq/L (5) 146 mEq/L (6) 150 mEq/L (7) 154 mEq/L	(1) Thermal Regulation Impairment, Central Sleep Apnea, GH/TSH/ACTH Deficiency, Seizures (2) Thermal Regulation Impairment, GH, TSH, ACTH Deficiency, Seizure, Sleep Impairment (3) Thermal Regulation Impairment, GH/TSH Deficiency, Seizure (4) None (5) Panhypopituitarism (6) GH/TSH/ACTH Deficiency, Thermal Regulation Impairment, Seizure (7) GH Deficiency	(1) Desmopressin Oral Melts 105 mcg/day (2) Desmopressin Oral Melts 15 mcg/day (3) Desmopressin oral Melts 85 mcg/day (4) Desmopressin Oral Melts 700 mcg/day (5) Stopped at age 2.5 (6) Never Treated (7) Stopped at age 3.6	(1) – (4) Not Described (5) Stopped Desmopressin at Age 2.5 Years (6) Self-resolved at 6 Months (7) Stopped at Age 3.6 Years
Crowley et al. 2007 [19]	Congenital Malformation, Not Further Described	n = 1	-	181 mEq/L	Panhypopituitarism, Sleep Apnea, DVT, Seizure	Desmopressin (Dosage Not Reported)	-
Zhang et al. 2018 [59]	Hypothalamic Hamartoma	n = 1	16 Years/Male	178 mEq/L	Seizure	Desmopressin 0.05mg BID (Route Not Described)	-
Modawi et al. 2013 [60]	Hypothalamic Astrocytoma	n = 1	20 Years/Male	180 mEq/L	Panhypopituitarism	Only Fluids	Deceased
Ramells et al. 2000 [61]	Hypothalamic Astrocytoma	n = 1	63 Years/Male	188 mEq/L	-	-	-
Elamin et al. 2020 [62]	Hypothalamic Astrocytoma	n = 1	12 Years/Female	173 mEq/L	Seizure, Obesity	Desmopressin 120 mcg BID oral	Deceased
Janus et al. 2014 [57]	Hypothalamic Astrocytoma	n = 1	17 Years/Male	163 mEq/L	TSH Deficiency, DVT, Seizure	Desmopressin (Dosage Not Reported)	Stopped Desmopressin
Hameed et al. 2012 [63]	Hypothalamic Astrocytoma	n = 1	16 Months/Female	156 mEq/L	Panhypopituitarism	DDAVP Subcutaneous 0.024mcg/dose BID	-
Laterre et al. 1969 [64]	Inflammatory Lesion of Hypothalamus	n = 1	21 Years/Male	163 mEq/L	Central Hypogonadism, Secondary Adrenal Insufficiency	Vasopressin	Deceased
Sridhar et al. 1974 [65]	Hypothalamic Tumour, Not Further Described	n = 1	17 Years/Female	190 mEq/L	Panhypopituitarism, Obesity	Chlorpropamide	
Behan et al. 2015 [35]	Hypothalamic Syndrome, Not Further Described	n = 1	-	-	-	-	-
Kavelaars et al. 2001 [66]	Non-Hodgkin’s Lymphoma with Hypothalamic Involvement	n = 1	48 Years/Male	173 mEq/L	Panhypopituitarism	Desmopressin 0.1mg	-
González Briceño et al. 2014 [67]	Hypothalamic Glioma	n = 1	26 Months/Female	-	-	-	-
O’Reilly et al. 2015 [68]	Neurosarcoidosis	n = 1	22 Years/Male	162 mEq/L	Anterior Hypopituitarism	Desmopressin (Dosage Not Reported)	Resolution with Infliximab
Crowley et al. 2007 [19]	Neurosarcoidosis	n = 1	33 Years/Male	162 mEq/L	Panhypopituitarism, Sleep Apnea, Seizure	Desmopressin (Dosage Not Reported)	-
Luciani et al. 1980 [69]	Neurosarcoidosis	n = 1	27 Years/Male	161 mEq/L	Central Hypogonadism, GH Deficiency	-	Deceased
Solis et al. 2020 [70]	Neurosarcoidosis	n = 1	22 Years/Female	180 mEq/L	Panhypopituitarism	Desmopressin (Dosage Not Reported)	-
Kumar et al. 2018 [71]	Langerhans Cell Histiocytosis	n = 1	36 Years/Female	156 mEq/L	Obesity, Thermal Regulation Impairment, Sleep Disorder, Seizure, Hypopituitarism (Hypothyroidism, Hypocortisolemia, Hypogonadism)	Desmopressin (Dosage Not Reported)	Deceased
Kaltsas et al. 2000 [72]	Langerhans Cell Histiocytosis	n = 1	46 Years/Male	-	Obesity, Sleep disorder, Short Term Memory Impairment, GH Deficiency, Central Hypogonadism	Desmopressin (Dosage Not Reported)	-
Tay et al. 2006 [73]	Langerhans Cell Histiocytosis	n = 1	22 Years/Male	-	Panhypopituitarism, Thermal Regulation Impairment	Desmopressin (Dosage Not Reported)	-
Avioli et al. 1962 [74]	Langerhans Cell Histiocytosis	n = 1	12 Years/Female	165 mEq/L	Central Adrenal Insufficiency	Vasopressin (Dose Not Described), Ultimately Discontinued with Just Fluids	Deceased
Yang et al. 2024 [33]	Langerhans Cell Histiocytosis	n = 6	-	-	-	-	-
Mendoza et al. 2015 [75]	Langerhans Cell Histiocytosis	n = 1	41 Years/Male	181 mEq/L	Secondary Hypogonadism, GH Deficiency	Desmopressin 0.1mg BID	-
Grimaldi et al. 1982 [76]	Langerhans Cell Histiocytosis	n = 1	Unknown Age/Female	-	-	-	-
Teelucksingh et al. 1991 [77]	Toluene Exposure	n = 1	14 Years/Male	166 mEq/L	Thermal Regulation Impairment, Sleep Apnea	Intranasal Desmopressin	-
Smith et al. 2002 [17]	Toluene Exposure	n = 1	26 Years/Male	-	Thermal Regulation Impairment, Sleep Apnea	-	-
Crowley et al. 2007 [19]	Toluene Exposure	n = 1	14 Years/Male	166 mEq/L	Sleep Apnea, Thermal Regulation Impairment, Seizure	Desmopressin (Dosage Not Reported)	-
Smith et al. 2002 [17]	Head Trauma	n = 1	22 Years/Male	-	-	-	-
Crowley et al. 2007 [19]	Head Trauma	n = 1	22 Years/Male	151 mEq/L	-	Desmopressin (Dosage Not Reported)	-
Thompson and Baylis 1987 [78]	Head Trauma	n = 1	22 Years/Male	-	-	Desmopressin (Dosage Not Reported)	-
Chua et al. 2021 [79]	Head Trauma	n = 1	70 Years/Male	166 mEq/L	Hypogonadotropic Hypogonadism	Oral Desmopressin 100mcg BID	-
Janus et al. 2014 [57]	Germinoma	n = 2	(1) 12 Years/Male (2) 8 Years/Female	(1) 176.6 mEq/L (2) 161 mEq/L	(1) Panhypopituitarism (2) Panhypopituitarism, DVT	(1) Desmopressin (Not Further Described) (2) Not Described	-
Trust et al. 1975 [80]	Authors Believe Either Germinoma or Pinealoma	n = 1	20 Years/Male	171 mEq/L	Seizure, Panhypopituitarism	Vasopressin Nasal (Dosage Not Reported)	-
Sklar et al. 1981 [81]	Germinoma	n = 2	(1) 11 Years/Male (2) 12 Years/Male	(1) 160 mEq/L (2) 170 mEq/L	(1) Gonadotrophin, GH and TSH Deficiency (2) Panhypopituitarism	Vasopressin (Dosage Not Reported)	-
Pomarede et al. 1982 [82]	8 patients with histologic proof of germinoma and adipsia, two without histologic proof	n = 8	(1) 12 Years/Female (2) 15 Years/Male (3) 12 Years/Male (4) 19 Years/Male (5) 11 Years/Male (6) 10 Years/Female (7) 14 Years/Male (8) 8 Years/Female	-	78% Hypopituitarism (Not Individually Reported)	-	-
Robertson 1984 [83]	Germinoma	n = 1	14 Years/Female	179 mEq/L	Seizure, Panhypopituitarism	Desmopressin (Dosage Not Reported)	-
Yang et al. 2024 [33]	Germinoma	n = 6	-	-	-	-	-
Lascelles and Lewis 1972 [41]	Germinoma	n = 1	15 Years/Female	163 mEq/L	Seizure	Vasopressin Spray	Deceased
Căpraru et al. 2014 [84]	Germinoma	n = 1	18 Years/Male	186 mEq/L	Sinus Thrombosis, DVT, Thermal Regulation Impairment, Central Hypogonadism, Hypothyroidism, Growth Hormone Deficiency	Desmopressin 10 mcg Nasal TID Desmopressin Acetate rhinal tube 0.1mL daily	
Arai et al. 1999 [85]	Germinoma	n = 1	17 Years/Male	160 mEq/L	-	Desmopressin	-
Kobayashi et al. 2019 [86]	Germ Cell Tumour	n = 1	11 Years/Male	183 mEq/L	Panhypopituitarism, Superior Sagittal Sinus Thrombosis	-	-
Sano et al. 1991 [87]	Germ Cell Tumour	n = 1	20 Years/Female	166 mEq/L	Panhypopituitarism, Thermal Regulation Impairment	3mcg Intranasal Desmopressin BID	-
Barraso et al. 2019 [37]	Mixed Germ Cell Tumour	n = 1	9 Years/Male	170 mEq/L	Panhypopituitarism	-	-
Bergadá et al. 2004 [88]	Mixed Germ Cell Tumour	n = 1	11 Years/Male	158 mEq/L	Panhypopituitarism, DVT	Intranasal Desmopressin 5 mcg Daily	
Pereira et al. 2015 [89]	Pinealoma	n = 1	13 Years/Female	-	Panhypopituitarism	Desmopressin Sublingual 0.72 mcg/day	-
Ball et al. 1997 [20]	Pinealoma	n = 1	15 Years/Male	166 mEq/L	Panhypopituitarism	Desmopressin (Dosage Not Reported)	-
Christie and Ross 1968 [90]	Ectopic Pinealoma	n = 1	18 Years/Female	166 mEq/L	Panhypopituitarism	Desmopressin Nasal 37.5 mg BID	
Dalan et al. 2019 [91]	Arteriovenous Malformation	n = 1	27 Years/Female	172 mEq/L	-	Desmopressin Melt oral 90 mcg qAM and 75 mcg qPM	-
Ball et al. 1997 [20]	Cavernous Hemangioma	n = 1	19 Years/Male	157 mEq/L	Panhypopituitarism, Short-Term Memory Impairment	Oral Desmopressin 100 mcg BID	-
Behan et al. 2015 [35]	Vascular Anomaly Not Further Described	n = 1	-	-	-	-	-
Hiyama et al. 2010 [92]	Autoantibodies to Sodium-Level Sensor in Brain	n = 1	6 Years/Female	199 mEq/L	-	No Desmopressin	-
Hiyama et al. 2017 [93]	Autoantibodies to Subfornical Organ	n = 3	(1) 9 Years/Female (2) 16 Years/Female (3) 8 Years/Female	(1) 155 mEq/L (2) 165 mEq/L (3) 161 mEq/L	-	(1) Percutaneous Desmopressin 4 mcg/dose (2) Desmopressin 5 mcg/day (3) Desmopressin 60 mcg/day oral	-
Sherlock et al. 2006 [94]	Macroprolactinoma	n = 1	14 Years/Male	157 mEq/L	Panhypopituitarism, Obesity, DVT, Sleep Apnea	Desmopressin Oral 200mcg BID	-
Ball et al. 1997 [20]	Non-Functioning Pituitary Adenoma	n = 1	14 Years/Female	160 mEq/L	Panhypopituitarism	Desmopressin 250mcg BID SC	
Sabzghabaei et al. 2018 [18]	Adenoma Resection	n = 1	68 Years/Female	175 mEq/L	-	Desmopressin (Dosage Not Described)	-
Arem et al. 1986 [95]	Non-Functioning Pituitary Adenoma	n = 1	39 Years/Male	149 mEq/L	Panhypopituitarism, Short Term Memory Impairment	0.1 mL Intranasal Desmopressin Every 24-54 hours, Chlorpropamide	-
Behan et al. 2015 [35]	Prolactinoma	n = 1	-	-	-	-	-
Miljic et al. 2014 [34]	Non-Functioning Pituitary Adenoma	n = 1	27 Years/Male	168 mEq/L	Secondary Hypogonadism, Obesity	Desmopressin	-
Lascelles and Lewis 1972 [41]	Pituitary Adenoma (Acromegaly)	n = 1	20 Years/Male	164 mEq/L	-	Desmopressin	-
Cherchir et al. 2024 [96]	Herpes Meningoencephalitis	n = 1	53 Years/Female	159 mEq/L	Central Hypothyroidism	Desmopressin 60 mcg BID	-
Barraso et al. 2019 [37]	Optic Nerve Glioma	n = 1	15 Years/Female	Not Described	Panhypopituitarism. Thermal Regulation Impairment	-	-
Latcha et al. 2011 [97]	Hepatocellular Carcinoma Metastases to Hypothalamus	n = 1	37 Years/Female	176 mEq/L	-	Desmopressin Oral 50 mcg BID	-
Lascelles and Lewis 1972 [41]	Lung Metastases to Hypothalamus	n = 1	62 Years/Male	169 mEq/L	Hypopituitarism	Desmopressin	Deceased
Fukagawa et al. 2001 [98]	Meningitis and Hydrocephalus	n = 1	32 Years/Female	166 mEq/L	-	-	-
Janus et al. 2014 [57]	Bacterial Meningitis	n = 1	10 Months/Female	170 mEq/L	TSH, ACTH Deficiency, Seizure	Desmopressin (Dosage Not Reported)	-
Janus et al. 2014 [57]	Optic Glioma Surgery	n = 1	16 Years/Male	171 mEq/L	ACTH Deficiency	-	-
Keuneke et al. 1999 [99]	Cytomegalovirus Encephalitis	n = 2	(1) 38 Years/Male (2) 42 Years/Male	(1) 164mEq/L (2) 162mEq/L	(1) Short-Term Memory Impairment, Thermal Regulation Impairment (2) None	(1) IV Desmopressin 4 mcg daily (2) Not Described	(1) Deceased (2) Deceased
Hu et al. 2013 [100]	Cytomegalovirus Infection	n = 1	12 Years/Female	166 mEq/L	-	Desmopressin 0.05 mg qAM, 0.025mg in afternoon and 0.05 mg in evening	
Bode et al. 1971 [31]	Third Ventricle Cyst	n = 1	8 Years/Female	174 mEq/L	Panhypopituitarism, Obesity	5 units Desmopressin IM every other day, Chlorpropamide	-
Verdin et al. 1985 [101]	Pseudotumour Cerebri/Empty Sella Syndrome	n = 1	30 Years/Female	163 mEq/L	Hypogonadotrophic Hypogonadism, Short-Term Memory Impairment	Acetazolamide	-
Lascelles and Lewis 1972 [41]	Suprasellar Meningioma	n = 2	(1) 61 Years/Female (2) 50 Years/Female	(1) 162 mEq/L (2) 160 mEq/L	(1) None (2) Hypopituitarism	(1) Desmopressin tannate 10 units every other day (2) Fluids only	-
Nandi et al. 1978 [30]	Unknown Cause	n = 1	24 Years/Male	184 mEq/L	Rhabdomyolysis, Renal Impairment	Chlorpropamide	-
Radetti et al. 1991 [47]	Unknown Cause	n = 1	3 Months/Female	173 mEq/L	-	Desmopressin Intranasal 0.05 mL BID	-
Villadsen and Pedersen 1987 [102]	Unknown Cause	n = 1	16 Years/Male	167 mEq/L	Obesity, Thermal Regulation Impairment, DVT	Nasal Desmopressin (Dosage Not Reported)	-
Behan et al. 2015 [35]	Infiltrative Condition, Not Further Described	n = 1	-	-	-	-	-
Yang et al. 2024 [33]	Pituitary Abscess, Hypophysitis, Rathk’'s Cleft Cyst, Prolactinoma, Epithelial Cyst, Hypothalamitis	n = 7	-	-	-	-	-
Green and Landt 2002 [103]	Unknown Causes	n = Unknown	-	Does not Describe Which Patients in the Study Have Adipsic Diabetes, nor Number of Patients Who Have Adipsia	-	-	-
Arima et al. 2014 [104]	Unknown Causes	n = 23 (11 Females, 12 Males)	-	Not Described Individually	Anterior Pituitary Dysfunction in n = 20	Desmopressin (Dosage Not Reported)	n = 4 Deceased
Firhan et al. 2021 [105]	Nasopharyngeal Metastases to Hypothalamus	n = 1	29 Years/Female	-	-	Desmopressin (Dosage Not Reported)	-
Magalhães et al. 2017 [106]	Idiopathic Thinning of Pituitary Gland and Stalk	n = 1	57 Years/Female	155 mEq/L	-	Desmopressin (Dosage Not Reported)	-
Lascelles and Lewis 1972 [41]	Unknown Cause	n = 1	42 Years/Female	160 mEq/L	Panhypopituitarism	Desmopressin	-
Lascelles and Lewis 1972 [41]	Optic Nerve Tumour Removal	n = 1	41 Years/Female	160 mEq/L	Panhypopituitarism	Vasopressin Tannate 1 unit Daily	-
Chothia et al. 2018 [107]	Idiopathic	n = 1	24 Years/Male	154 mEq/L	Polycythemia	No Desmopressin, Only Fluids	-
Massot et al. 1977 [108]	Idiopathic	n = 1	13 Years/Female	-	-	-	-
Brezis and Weiler-Ravell 1980 [109]	Idiopathic	n = 1	80 Years/Female	158 mEq/L	Panhypopituitarism	-	-

Adipsic diabetes insipidus occurs when there is significant damage in the hypothalamus at the areas that make vasopressin, and the thirst centres. Adipsic diabetes insipidus has been described following anterior communicating artery aneurysm repairs, intracranial tumors (craniopharyngioma, pinealoma, germinoma, pituitary tumors), head trauma, vascular malformations, infiltrative diseases, and congenital lesions among others; a full list is depicted in Table 3 [110]. The most common cause of adipsic diabetes insipidus is following treatment of a ruptured anterior communicating artery aneurysm (responsible for around 40% of cases) [91]. The risk of developing the condition following repair of an anterior communicating artery aneurysm is around 0.04% (with meta-analyses noting coiling associated with better outcomes over clipping) [16]. Following aneurysmal ruptures, the next most common causes include craniopharyngiomas (13-30%) and congenital causes (5-20%) [62]. As demonstrated in the study by Mavrakis and Tritos [6], patients with adipsic diabetes insipidus and a ruptured anterior communicating artery aneurysm are significantly likely to be older than those with craniopharyngiomas, germinomas, or congenital condition (age 39.8 +/- 3.2 versus 23.1 +/- 4.9 versus 13.3 +/- 1.0 versus 5.5 +/- 2.4 years, p < 0.001).

**Table 3:** Causes of Adipsic Diabetes Mellitus Adapted with permission from [110]

Causes of Adipsic Diabetes Insipidus
Repair of Ruptured Anterior Communicating Artery Aneurysm
Craniopharyngioma
Pinealoma
Germinoma
Pituitary Adenoma (Macroprolactinoma, Non-Functioning Adenoma)
Toluene Exposure
Neurosarcoidosis
Head Trauma
Autoantibody Formation
Arteriovenous Malformation/Cavernous Hemangioma
Cytomegalovirus Encephalitis
Langerhans Cell Histiocytosis
Pseudotumour Cerebri
Congenital Lesions: Septo-Optic Dysplasia; Encephalocele; Holoprosencephaly; Agenesis of Septum Pellucidum; Dysgenesis of Corpus Callosum

Pathophysiology

Water balance is maintained by vasopressin release and the sensation of thirst. Changes in osmolality are among the most powerful triggers for the production and release of vasopressin, alongside thirst, to control a tight osmolality of the serum between 285-295 mOsm/kg [110]. As well as osmolality, baroreceptor response to both pressure and volume status are factors inducing the release of vasopressin [110].

**Figure 2:**
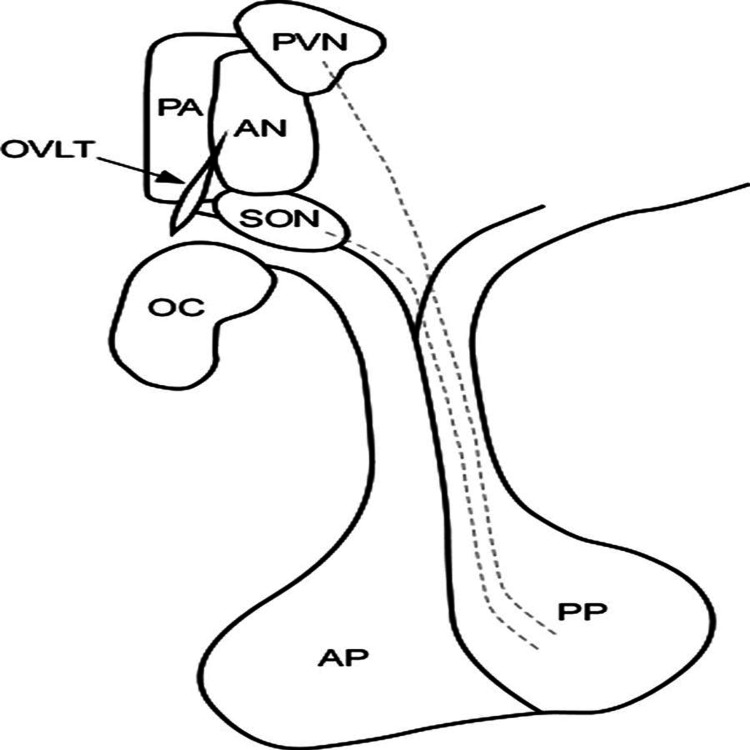
Nuclei of Water Balance Obtained with Permission from [6] AP: Anterior Pituitary; OC: Optic Chiasm; OVLT: Organ Vasculosum of Lamina Terminalis; PA: Preoptic Area; PP: Posterior Pituitary; PVN: Paraventricular Nuclei; SON: Supraoptic Nucleus

Osmoreceptors are located within the organum vasculosum of the lamina terminalis (OVLT), the subfornical organ and the anterior hypothalamus, with the nearby vasopressin-producing cells being located in the paraventricular and supraoptic nuclei of the hypothalamus (extending along axons into the pituicytes) (Figure 2). The osmoreceptors within the OVLT are close to the anterior wall of the third ventricle and are therefore readily able to detect such changes and respond appropriately [16]. Following the release of vasopressin, it binds to V_2_ receptors in the collecting duct of the renal tubule, leading to aquaporin-2 insertion within the luminal membrane, allowing for water permeation and reabsorption from urine into the blood, alongside stimulation of thirst centers within the hypothalamus. Due to such a tightly formed pathway, it is estimated that osmolality varies by no more than 2% in the setting of unrestricted access to water [111].

Blood supply of the OVLT is from four predominant sources, including superior branches of the anterior communicating artery, two lateral branches from the anterior cerebral arteries (itself branching from the anterior communicating arteries), and an inferior branch that ascends from below the optic chiasm [91]. As a result, interventions such as clipping and coiling lead to downstream interruption and can result in adipsia and abnormalities in both vasopressin synthesis and release.

Postoperative diabetes insipidus is a common finding after pituitary surgery characterized by a triphasic response of hypothalamic dysfunction and early diabetes insipidus (with polyuria and hypernatremia), syndrome of inappropriate antidiuretic hormone secretion (SIADH) due to release of pre-formed vasopressin from storage vesicles in the posterior pituitary, with a return to permanent diabetes insipidus due to depletion of these stores [110]. With injury to the hypothalamic thirst centers, adipsia may remain alongside the central diabetes insipidus. Adipsic disorders are classified into four subtypes, A through D, with the patient described in the case report presenting with Type C Adipsia (Table 4) [62,112,113].

**Table 4:** Subtypes of Adipsia Collated from [62,112,113]

Subtype	Description
Type A	Also termed ‘Essential Hypernatremia’. Occurs from reduced sensitivity of the osmoreceptors, leading to partial diabetes insipidus with concurrent subnormal sensation of thirst. It is characterized by an upward reset of the osmotic threshold for both thirst and vasopressin release (until osmolality has risen above the new threshold (often over 300 mOsm/Kg)). Patients are often protected from extremes of hypernatremia as a result. With water overload, thirst can be suppressed alongside vasopressin release, and hypotonic diuresis can develop. Underlying etiology is unknown as imaging of the pituitary is often unremarkable.
Type B	Subnormal vasopressin and thirst response to rising osmotic stimuli, with normal osmoregulatory point. Hypothesized to be a result of partial damage of the osmoreceptors. Described in patients with microcephaly and dysplastic corpora callosa. Baroreceptor-regulated vasopressin release is often intact, as is the release in response to insulin-provoked hypoglycemia and emesis. Appears to be increased renal sensitivity to vasopressin, and patients may retain capability of limiting free water excretion by concentrating the urine.
Type C	Described as the complete absence of thirst response and release of vasopressin with rising osmotic stimuli, caused from central impairment (such as clipping of anterior communicating artery aneurysm). Present with lack of thirst and polyuria. Often considered the most difficult subtype to manage, and patients are at risk for life-threatening hypernatremia.
Type D	Thirst response is absent but osmoregulation of the release of vasopressin is maintained. Only a single case has been described in the literature.

Diagnosis

**Figure 3:**
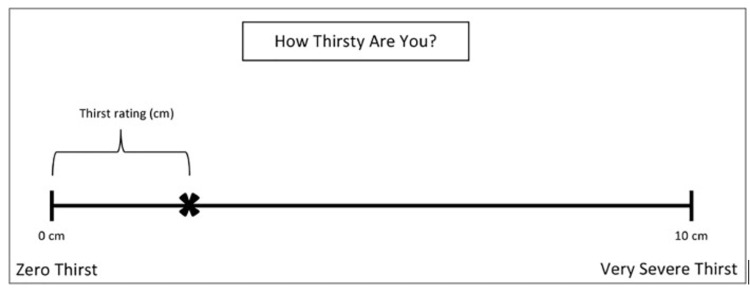
Visual Analog Scale of Thirst Obtained with Permission from [110]

Adipsia is diagnosed when thirst is absent and spontaneous consumption of water does not occur with both hypernatremia (above 150 mEq/L) and hyperosmolality (above 310 mOsm/Kg) [110]. Furthermore, water deprivation tests (or hypertonic stimuli such as saline infusions) can be performed, assessing consumption of water afterwards (lower consumption is consistent with adipsic diabetes insipidus [110]). A visual analog scale is frequently encountered in both clinical practice and research for a subjective assessment of thirst (Figure 3) [110]. Crowley et al. [19] demonstrated significantly less water consumed (and less thirst sensation) in adipsic patients compared to controls, following the administration of a hypertonic saline infusion (p < 0.0017 and p < 0.001, respectively). The authors additionally note the depressed release of vasopressin following hypertonic saline solution administration compared to controls (p < 0.001).

Although patients with adipsic diabetes insipidus have impaired vasopressin and thirst response to increasing osmolality, the response to hypotension and nausea can be varied, and appears to be dependent on the underlying cause (and likely extent of cerebral involvement). Notably, in patients following resection of a craniopharyngioma, the response to hypotension is absent, but usually preserved following coiling of a ruptured anterior communicating artery aneurysm. This was demonstrated by Smith et al. [17], whereby nine patients with adipsic diabetes insipidus were compared to controls. As expected, hypertonic saline infusion produced absent thirst and vasopressin response in the adipsic group compared to the control group (p < 0.001). Subsequently, trimetaphan was infused to produce a drop in the mean arterial pressure in both groups; none of craniopharyngioma patients (n = 3) demonstrated a significant rise in vasopressin (however, there was a significant rise in the remaining n = 6, p < 0.001). Mavrakis and Tritos [6] further parallel these findings in 13 out of 20 patients by assessing vasopressin response to baroreceptor-mediated challenges or emesis (from apomorphine), again demonstrating that patients with craniopharyngiomas were less likely to respond (p < 0.01). Crowley et al. [19] additionally demonstrated that trimetaphan failed to lead to vasopressin release in all craniopharyngioma patients (n =4) as well as one patient with a macroprolactinoma, but the response was preserved in the remaining patients with adipsic diabetes insipidus (n = 9) (p < 0.008).

Complications

Patients with adipsic diabetes insipidus are at risk for numerous comorbidities, including hypernatremia, hyponatremia, venous thromboembolism, seizures, acute kidney injury, rhabdomyolysis, obesity, sleep-disordered breathing, anterior pituitary hormone deficiencies, hypothalamic disruption, and an overall increase in mortality [21].

Arima et al. [104] note a relative risk of 68 for hypernatremia (described as a serum sodium level of above 150 mEq/L) in patients with adipsic diabetes insipidus compared to those with central diabetes insipidus with intact thirst (p < 0.001). Whilst patients may be asymptomatic, the severity of the hypernatremia is often an indication for recurrent hospitalizations consisting of days to weeks, compared to those with an intact thirst sensation. Yang et al. [33] note that patients with adipsic diabetes insipidus are likely to have longer hospital durations compared to controls (9.6 days compared to 5.9 days, p = 0.000). Of note, however, as noted by Elamin et al. [62], such patients are often paradoxically at risk of hyponatremia due to the inability to appreciate fluid overload when adherent to oral hydration and desmopressin administration.

It is suggested that seizures are due to the presence of hypernatremia; however, eunatremic seizures are noted in around 50% of patients; in the study by Yang et al. [33], however, there was no significant difference in rates of seizures between adipsic patients and controls. While rhabdomyolysis can occur from seizures, more commonly, it is due to osmolar disturbances from fluid shifts (and subsequent muscle rupture), which can lead to acute kidney injury due to tubular injury from myoglobin. In addition to the rhabdomyolysis, patients are prone to acute kidney injury due to dehydration causing renal vasoconstriction; the study by Yang et al. [33] noted a significant risk of renal insufficiency compared to controls (12% versus 1%, p = 0.002).

Patients with adipsic diabetes insipidus compared to central diabetes insipidus have an odds ratio of 8.8 for the development of serious infections (p < 0.001), notably respiratory tract, reproductive systems and wounds along the abdomen; the causation is not readily known but appears to be related to recurrent hospitalizations [104].

Yang et al. [33] note higher rates of hyperglycemia, dyslipidemia and hyperuricemia in such patients; however, after multivariate logistic regression, only hyperglycemia appears to be significant with an odds ratio of 5.886 (p = 0.003). The authors further suggest a higher risk of venous thrombosis (14% versus 1%, p = 0.002), including the lower extremities and cerebral venous sinuses. This finding was first reported by Crowley et al. [19] and is likely related to the underlying state of dehydration, as large cohort studies note this is more frequent when thirst is not intact. Moreover, it is likely that the prolonged hospitalizations account for the heightened risk for deep venous thromboses due to related immobility. Another consideration is the treatment itself (desmopressin), which can lead to the release of factor VIII and von Willebrand factor from endothelial cells; however, it is noted that this would be clinically significant with a dose ten-fold of what is administered in such patients [110]. A final consideration is the concomitant finding of sleep-disordered breathing in adipsic patients, whereby sleep apnea (present in more than 50% of patients in one case series) may contribute to polycythemia and predispose to a hypercoagulable state [19].

Sleep disordered breathing in patients with adipsic diabetes insipidus includes both obstructive and central sleep apnea, as well as obesity-related hypoventilation. It appears that patients with craniopharyngiomas have a higher obesity apnea-hypopnea index and demonstrate reductions in oxygen saturation overnight [110]. Moreover, in patients with craniopharyngiomas, there is a greater chance of hypothalamic injury, which itself can be associated with sleep-disordered breathing. Adults with craniopharyngiomas have also been noted to have excessive daytime sleepiness, persisting even when matched for controls [110]. Cognitive dysfunction is a common finding associated with adipsic diabetes insipidus, including fatigue, short-term memory loss, and behaviour disturbances, which can lead to poor adherence with the respective treatment regimen.

Adipsic diabetes insipidus due to craniopharyngiomas is commonly associated with other hypothalamic disturbances, such as appetite disturbances (food cravings and over-eating), temperature instability, and behavioural misconduct [110]. Notably, around 20-46% of patients with adipsic diabetes insipidus are either overweight or obese, most often described in the adult population [19, 110]. Additionally, as with our patient, anterior pituitary function can be compromised, ranging from single defects to panhypopituitarism. Mavrakis and Tritos [6] note 72% of their mixed cohort had deficits in anterior pituitary function, with 44% demonstrating panhypopituitarism, and 28% with partial defects in hormonal secretion; the authors, however, note this is significantly less likely in patients with anterior communicating artery aneurysm ruptures compared to craniopharyngiomas (p < 0.001).

Patients with adipsic diabetes insipidus also have a heightened premature death rate. Arima et al. [104] demonstrate an adjusted odds ratio of mortality for such patients to be 9.53 (95% CI 1.85-40.08, p = 0.007), most related to infection and respiratory failure (sleep-disordered breathing and venous thromboembolism).

Treatment

With the significant risks of comorbidities and mortality, as noted above, adherence to treatment is of utmost importance. Recommendations for treatment involve close monitoring of fluid intake (and documenting throughout the day), which is typically advised to be no more than 1.5-2 liters (titrated based on follow-up results). Patients are advised to pay close attention to insensible losses (such as from fever, diarrhea, exercise, warmer climates) which will require an increase in oral hydration [27]. Patients are weighed when eunatremic to determine the target weight, followed by which they are weighed daily; if underweight, then the subtraction of the desired and current weight is added in oral hydration as liters. Over time, patients can be prescribed a ‘sliding scale’ for oral hydration based on body weight [110].

Regular sodium monitoring is recommended, while there are no formal guidelines, Eisenberg and Frohman [110] recommend weekly sodium monitoring. Although primitive, home sodium capillary monitoring devices have been developed and trialed in both adults and children, accurately measuring sodium from capillary blood (r = 0.92 correlation between capillary and laboratory sodium), which can be incorporated into the customized oral hydration sliding scale [103,110].

Patients are furthermore prescribed desmopressin (DDAVP) to achieve a urine output of 1.5-2 liters daily. The dosage of desmopressin varies, as well as formulation (oral: 100- 250 micrograms twice daily; intranasal 3-10 micrograms twice per day) [62,63]. Oral absorption is often less preferable due to the significant variation with individuals and must be taken on an empty stomach [110]. In neonates, some recommended injection therapy to minimize the absorption problems [110]. Patients are often advised to avoid alcohol, due to the rapid and large shifts in fluid status that can occur [113].

Due to the correlation with memory deficits in certain patients with adipsic diabetes insipidus (which appears to be multifactorial), family-assisted reminders and prompts are beneficial. Behavioral modification, relying on scheduled hydration based on daily weights, urine output and laboratory monitoring, as well as reward systems, has proven successful [29].

Within the literature, anecdotal reports of chlorpropamide (a first-generation sulfonylurea medication) increasing awareness of thirst have been described, however, this is matched with the risk of hypoglycemia, hepatic injury, hematologic abnormalities and dermatological reactions [31]. Similarly, limited reports note enhanced antidiuresis following the administration of clofibrate [21]. These medications appear to induce SIADH and are not recommended due to the unpredictable side effects and variable responses. 

Infrequently, patients may regain either partial, or complete recovery of thirst perception, months-to-years following the initial insult. Our patient appeared to regain partial thirst sensation approximately four months after the treatment of the ruptured aneurysm. Whilst thirst response may improve, the response of vasopressin to the increase in osmolality remains altered.

## Conclusions

Adipsic diabetes insipidus is characterized by lack of vasopressin response to hyperosmolality and hypernatremia, with impairment of thirst sensation. While numerous causes in the literature have been depicted, the most common cause (as with our case report) is following coil embolization of a ruptured communicating artery aneurysm. Compared to central diabetes insipidus with preserved thirst sensation, adipsic patients are at heightened risk for comorbidities and mortality from the inability to appreciate thirst. Although some patients (as with our case report) may develop variable recovery in thirst sensation, it is not possible to predict which patient will regain this sensation. Treatment typically requires patient education, fixed desmopressin dosages, frequent osmolality monitoring, titration of urine output, and control of the amount of fluid intake (with corrections for insensible losses). This case report and literature review provide an overview of the typical presentation and management, alongside a review of the current literature pertaining to adipsic diabetes insipidus.
